# Acknowledgement to Reviewers of *Dentistry* *Journal* in 2018

**DOI:** 10.3390/dj7010007

**Published:** 2019-01-18

**Authors:** Dentistry Journal Editorial Office

**Affiliations:** MDPI, St. Alban-Anlage 66, 4052 Basel, Switzerland

Rigorous peer-review is the corner-stone of high-quality academic publishing. The editorial team greatly appreciates the reviewers who contributed their knowledge and expertise to the journal’s editorial process over the past 12 months. In 2018, a total of 67 papers were published in the journal, with a median time to first decision of 18.8 days and a median time to publication of 43 days. The editors would like to express their sincere gratitude to the following reviewers for their cooperation and dedication in 2018:

Aartman, Irene Helena AdrianaLaskin, DanielAbdelaziz, MarwaLauritano, DorinaAbrams, StephenLeite, Fábio R.M.Agrawal, PriyankaLevesque, CelineAksel, HacerLittle, JulianAlhalawani, AdelLiu, YuAlmståhl, AnnicaLoewy, ZviAntoniadou, MariaLópez Jornet, PiaArdelean, Lavinia CosminaŁosiewicz, BożenaArora, HimanshuLukomska-Szymanska, MonikaAssari, ShervinLuongo, GiuseppeAvent, MinyonMaiorana, CarloBaghdadi, Ziad D.Mante, Francis K.Bagnato, Vanderlei SalvadorManton, David J.Bakr, Mahmoud M.Marshman, ZoeBanas, JeffreyMartino, SabataBaruth, AndrewMartins, Ian JamesBatchelor, PaulMathur, ManuBecker, KathrinMatys, JacekBerg, Britt-IsabelleMeister, JörgBernardi, SaraMilardović, SlađanaBian, LiMine, AtsushiBlastname, FirstnameMorita, ManabuBlum, Igor RMuthaiyan, ArunachalamBoffano, PaoloMystkowska, JoannaBortolotto, TissianaNalliah, RomeshBorzabadi-Farahani, AliNicholson, John W.Bourgeois, DenisNieminen, PenttiBourgeois, DenisPalomo, LeenaBrowar, Andrew W.Papaccio, GianpaoloBullon, PedroPapantonopoulos, GeorgeCarey, CliftonPenarrocha, MiguelCayón De Las Cuevas, JoaquínPepelassi, EudoxieChass, GregoryPilloni, AndreaChen, Min-HueyPirani, ChiaraChinelatti, MichellePommer, BernhardChiquet, Brett T.Porritt, JennyChladek, GrzegorzPrados-Frutos, Juan-CarlosChu, Chun-HungQuock, RyanCicciù, MarcoRanzato, EliaCicciù, MarcoReiter, E. MirandaCionca, NorbertRios, Jose VicenteClastname, FirstnameRobertson, DouglasColeman, NicholaRodd, HelenColetta, Ricardo D.Rodella, FabrizioCollins, Maurice N.Roggendorf, Matthias JCrocombe, LeonardRoh, SanghoCsík, AttilaRyabov, IgorCuller, CorinnaSafavi, KamranCurtis, DeniceSagheb, KeyvanCvikl, BarbaraSahrmann, PhilippCvikl, Doz. BarbaraSalehi, Hassan S.Dammaschke, TillSandy, Jonathan RDanesh-Sani, Seyed AmirSantos, João M.Darvell, BrianSarmast, Nima D.De Hemptinne, FerdinandSasaki, Jun-IchiDeinzer, RenateSato, TakuichiDlastname, FirstnameSawai, HirofumiDo, ThuyScribante, AndreaDonchin, MilkaSefat, FarshidDorn, Samuel O.Seligman, Laura D.Duryee, Michael J.Shin, Jae M.Ekuni, DaisukeShort, LeonieEl-Bialy, TarekSigusch, Bernd W.Elgazzar, RedaSilikas, NickEl-Housseiny, AzzaSilikas, NikolaosFareed, AmberSing, Swee LeongFarronato, DavideSmith, Leonard B.Fauzi, IlianaSong, Linyong (Leon)Felix Gomez, Grace GomezSpagnuolo, GianricoFerreira, Manuel MarquesSperber, Geoffrey H.Fine, James BurkeSpinas, EnricoFiner, YoavStan, Miruna SilviaForner, LeopoldoSugiyama, MasaruFujihara, HisakoSwann, Brian JeffreyGalluccio, GabriellaTallarico, MarcoGettleman, LawrenceTanaka, YutoGorseta, KristinaTchan, Michel C.Goss, Alastair N.Tham, RachelGray, AndrewThikkurissy, SaratGregory, Richard LTodea, Darinca CarmenHee, Ay ChingTorres Lagares, DanielHegedűs, CsabaTraini, ToninoHeima, MasahiroTroedhan, AngeloHemphill, Jean CroceTsoi, JamesHorowitz, Robert A.Tsuchida, SachioHowe, BrianTulunoglu, IbrahimHristov, IlianUpshur, Carole CIsola, GaetanoVan Merkesteyn, J.P. RichardItri, AngeloVaz, Irene PinaIwata, JunichiVeerkamp, JaapJAMSHED, SHAZIAVettore, Mario VJefferies, Steven R.Vozza, IoleJeng, Jiiang-HueiWalsh, Laurence JKaisarly, DaliaWalter, ChristianKalenderian, ElsbethWang, Cheng-PingKavvadia, KaterinaWang, Jeff CW.Kellesarian, SergioWieckiewicz, MieszkoKhaled, YasserWierichs, RichardKim, Jee HwanWijesinghe, Ruchire ErangaKim, Jin-BomWikström, MaudeKitamura, ChiakiWillaert, EvaKoizumi, HiroyasuXi, WeixianKooyman, David L.Yamamoto, YusukeKorsunsky, Alexander M.Yoshida, KenjiKosuke, NozakiYuan, KuoKranz, StefanYumoto, HiromichiKujan, OmarZandona, Andrea FerreiraKumar, SunilZhan, LingKuo, Tzong-FuZhang, RuiyongKurien, Biji TheyilamannilZhao, LinpingKwon, Tae-YubŻmudzki, JarosławLahiri, AmitabhaZoidis, PanagiotisLam, Otto Lok Tao


